# Genotype-Specific HPV E6/E7 mRNA Triage Improves Risk Stratification and Reduces Referrals in DNA-Positive ASC-US/LSIL: A Real-World Cohort from Nordland, Norway

**DOI:** 10.3390/pathogens15020178

**Published:** 2026-02-06

**Authors:** Khalid Al-Shibli, Dat Tan Nguyen, Hiba Abdul Latif Mohammed, Sveinung Wergeland Sørbye

**Affiliations:** 1 Department of Pathology, Nordlandssykehuset HF, 8092 Bodø, Norway; khalid.al-shibli@nordlandssykehuset.no (K.A.-S.); dat.tan.nguyen@nordlandssykehuset.no (D.T.N.); 2 Department of Gynecology, Nordlandssykehuset HF, 8092 Bodø, Norway; hiba.abdul.latif.mohammed@nordlandssykehuset.no; 3 Department of Clinical Pathology, University Hospital of North Norway, 9019 Tromsø, Norway

**Keywords:** cervical cancer screening, HPV DNA, ASC-US, LSIL, HPV E6/E7 mRNA, genotype-specific triage, CIN2+, CIN3+, extended HPV genotyping, colposcopy referral, PreTect HPV-Proofer 7

## Abstract

HPV DNA–positive women with ASC-US/LSIL cytology represent a heterogeneous risk group in cervical screening and require efficient triage. We evaluated a genotype-specific 7-type HPV E6/E7 mRNA assay (PreTect HPV-Proofer 7; types 16/18/31/33/45/52/58) in a real-world quality-assurance cohort at Nordland Hospital (Bodø, Norway). Among HPV-positive women with ASC-US/LSIL reflex cytology, 225 had sufficient residual liquid-based cytology material and a valid mRNA result; 175 had complete follow-up (2022–2025) and were included. Overall, 44.6% (78/175) were mRNA-positive (ASC-US 45.2%; LSIL 43.3%). For CIN2+, sensitivity was 63.4%, specificity 61.2%, PPV 33.3%, and NPV 84.5%; CIN2+ risk was 33.3% in mRNA-positive versus 15.5% in mRNA-negative women (RR 2.16, 95% CI 1.23–3.78). For CIN3+, risk was 14.1% versus 6.2%. Genotype-specific PPVs were highest for HPV33, HPV18, HPV16, and HPV31. In a referral simulation, mRNA-guided triage reduced baseline colposcopy referrals by 55% and decreased colposcopies per detected CIN2+ by ~30%, while 15 CIN2+ and 6 CIN3+ occurred in the mRNA-negative group and would be expected to be detected at 12-month follow-up among women with persistent HPV positivity. Genotype-aware HPV E6/E7 mRNA triage improves risk stratification and may increase screening efficiency.

## 1. Introduction

Cervical cancer prevention relies on early detection and treatment of high-grade cervical intraepithelial neoplasia (CIN2+) [1]. In recent years, many screening programs have transitioned from cytology to human papillomavirus (HPV) DNA testing as the primary method because of its superior sensitivity for precancerous lesions [2,3]. However, HPV DNA testing has lower specificity, detecting numerous transient infections that do not progress to disease. Effective triage of HPV DNA–positive women, therefore, remains critical to maintain clinical efficiency and avoid unnecessary procedures [4,5,6,7,8].

In Norway, liquid-based cytology (LBC) continues to serve as the standard triage following a positive HPV DNA screen. Women with atypical squamous cells of undetermined significance (ASC-US) or low-grade squamous intraepithelial lesions (LSIL) are commonly referred to colposcopy, although the risk of underlying CIN2+ in these groups is highly heterogeneous and most abnormalities regress spontaneously [9]. A more specific molecular triage marker could reduce overtreatment while maintaining safety.

As of 2025, the updated Norwegian Cervical Cancer Screening Programme (NCCSP) guidelines have introduced extended HPV DNA genotyping as the basis for triage in all HPV-positive women aged 25–69 years. Under the new framework, HPV DNA primary screening must include genotyping prior to cytology assessment—either by extended genotyping assays or, when partial-genotyping assays are used, through reflex testing of all HPV DNA–positive samples to determine genotype. Women positive for HPV16 are referred directly to colposcopy and biopsy regardless of cytological findings, whereas those positive for HPV18, 31, 33, 45, 52, or 58 and presenting with ASC-US or LSIL cytology are also referred for immediate colposcopic evaluation. Women positive for any of the remaining seven high-risk HPV types (35, 39, 51, 56, 59, 66, 68) are scheduled for repeat HPV testing after three years (ages 25–29) or one year (ages ≥ 30) [10].

While extended DNA genotyping improves risk discrimination by identifying infection with the most oncogenic HPV types, DNA testing alone cannot distinguish between transient infections and those undergoing transcriptionally active oncogenic transformation [11,12,13]. This limitation highlights the potential complementary value of mRNA-based triage within DNA-positive groups.

HPV E6/E7 messenger RNA (mRNA) detection identifies transcriptionally active infections that drive oncogenic transformation, providing a biologically relevant indicator of disease risk [14,15]. The genotype-specific 7-type HPV E6/E7 mRNA test (PreTect HPV-Proofer 7; detecting types 16, 18, 31, 33, 45, 52, and 58) builds upon earlier studies using the 5-type version of the assay (PreTect HPV-Proofer), which demonstrated higher specificity and positive predictive value (PPV) than HPV DNA testing or cytology in the triage of women with low-grade cytological abnormalities. More recent population-based data from northern Norway, employing the 7-type assay, further confirmed that mRNA triage of HPV DNA–positive women reduces referral rates and improves the efficiency of CIN2+ detection compared with cytology [16].

The clinical risk associated with HPV infection varies substantially by genotype [12]. Persistent infection with HPV16 and HPV18 accounts for ~70% of cervical cancers worldwide, and together with HPV31, HPV33, HPV45, HPV52, and HPV58 they account for >90% of cases [17]. Notably, HPV16, HPV18, and HPV45 are responsible for the majority of cervical cancers diagnosed in younger women and exhibit faster progression rates than other high-risk genotypes [18,19]. Recognizing this heterogeneity is essential for triage, as genotype-specific assessment can distinguish women at the highest short-term risk of CIN2+ from those with transient or low-risk infections.

Incorporating genotype-specific mRNA detection, therefore, offers a biologically grounded strategy for refining risk stratification within HPV DNA–positive women, including younger women who have a high prevalence of HPV infection and high CIN2 detection rates but also substantial potential for spontaneous regression. mRNA triage may help identify which infections are clinically meaningful and which may safely be monitored.

The current study evaluated the performance of genotype-specific HPV E6/E7 mRNA triage in a real-world quality-assurance cohort at Nordland Hospital, Bodø. Specifically, we assessed mRNA positivity, diagnostic accuracy (sensitivity, specificity, PPV, NPV), genotype-specific risks for CIN2+/CIN3+, and potential reductions in referrals compared with referring all HPV DNA–positive ASC-US/LSIL cases to colposcopy.

## 2. Materials and Methods

### 2.1. Study Design, Setting, and Population

This single-center, quality-assurance cohort study was conducted at Nordland Hospital, Bodø, Norway. Consecutive women participating in the Norwegian Cervical Cancer Screening Programme who tested positive for HPV DNA on primary screening and had low-grade cytology (ASC-US or LSIL) were eligible for inclusion. Women were enrolled between 1 January 2022 and 31 December 2024, and a clinical follow-up was completed on 31 August 2025.

**Figure 1:**
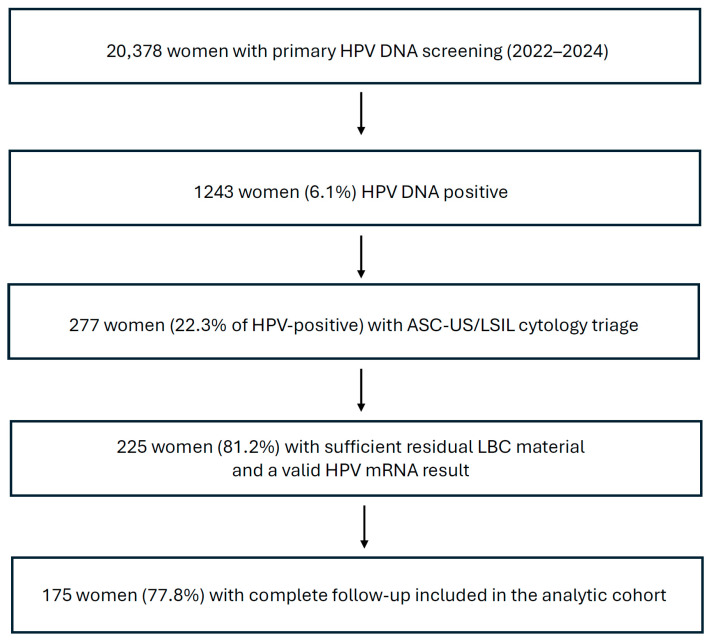
Flow diagram of the study population. During 2022–2024, 20,378 women underwent primary HPV DNA screening; 1243 (6.1%) were HPV DNA positive, and 277 (22.3%) had ASC-US/LSIL cytology triage. Of these, 225 women had sufficient residual liquid-based cytology (LBC) material available for HPV mRNA testing and were therefore eligible for inclusion. The final analytic cohort comprised 175 women with complete follow-up, defined as histology (biopsy) and/or a documented HPV-negative follow-up test within the observation period. Exclusions were due to insufficient residual LBC material for mRNA testing (*n* = 52) and incomplete follow-up (no histology and no documented HPV-negative follow-up test within the observation period; *n* = 50). Abbreviations: HPV, human papillomavirus; DNA, deoxyribonucleic acid; mRNA, messenger ribonucleic acid; LBC, liquid-based cytology; ASC-US, atypical squamous cells of undetermined significance; LSIL, low-grade squamous intraepithelial lesion.

During the study period, 20,378 women underwent primary HPV DNA screening; 1243 (6.1%) were HPV DNA–positive. Among the HPV-positive women, 277 (22.3%) had ASC-US/LSIL cytology at triage. Of these, 225 women had sufficient residual liquid-based cytology (LBC) material available for HPV mRNA testing and were therefore eligible for inclusion. The final analytic cohort comprised 175 women (ASC-US, *n* = 115; LSIL, *n* = 60) with complete follow-up, defined as histology (biopsy) and/or a documented HPV-negative follow-up test within the observation period. Exclusions from the analytic cohort were due to insufficient residual material for mRNA testing (*n* = 52) and incomplete follow-up (*n* = 50) (Figure 1).

### 2.2. Screening Tests and Triage Workflow

Primary HPV testing was performed using the cobas^®^ 4800 HPV Test (Roche Molecular Systems, Pleasanton, CA, USA) according to the manufacturer’s instructions. The assay qualitatively detects HPV DNA, providing type-specific results for HPV16 and HPV18 and a pooled result for 12 additional oncogenic types (31, 33, 35, 39, 45, 51, 52, 56, 58, 59, 66, and 68). Cervical specimens were clinician-collected and placed in PreservCyt^®^ medium (ThinPrep^®^, Hologic, Marlborough, MA, USA). Reflex liquid-based cytology (LBC) was performed on residual material from the same LBC specimen for all HPV DNA–positive samples; thus, there was no interval loss-to-follow-up between the HPV screening result and the cytology outcome within this clinician-collected pathway. Cytology was classified using Bethesda terminology [20]. Women with ≥ASC-US were managed according to national guidelines, with colposcopy and biopsy at the clinician’s discretion. Self-sampling is available in Norway for selected non-attenders, but self-collected samples were not included in this study.

To provide objective risk stratification within this DNA-positive, low-grade subset, all cases were further analyzed with a genotype-specific E6/E7 mRNA assay (PreTect HPV-Proofer’7, PreTect AS, Klokkarstua, Norway), performed on residual material from the same PreservCyt^®^ specimen. The assay targets transcripts from HPV16, 18, 31, 33, 45, 52, and 58 and detects E6/E7 mRNA, indicating transcriptional activity of viral oncogenes that are central to cervical carcinogenesis and thus serve as markers of biologically relevant, potentially transforming HPV infections. The test has demonstrated higher clinical specificity than HPV DNA testing for triage of ASC-US and LSIL in multiple cohorts [14,15,21,22,23,24,25,26].

### 2.3. Definitions and Endpoints

The primary endpoint was CIN2+, and the secondary endpoint was CIN3+. Histological endpoints were determined from routine pathology reports within the observation period; when multiple histologic outcomes were available for a participant, the highest-grade diagnosis was used. Cytology results followed Bethesda terminology, and HPV DNA and mRNA test outcomes were classified dichotomously (positive/negative). Women without histology were classified as having no CIN2+ if they had a documented negative HPV DNA test at follow-up within the observation period, consistent with the routine follow-up algorithm for triage-negative women. Women with insufficient follow-up to classify outcome (i.e., neither histology nor a documented HPV-negative follow-up test within the observation period) were excluded from the analytic cohort.

For genotype-specific analyses of the mRNA assay, each woman was assigned a single index genotype when more than one mRNA type was detected. Assignment followed a pre-specified hierarchy based on established oncogenic potential (16 > 18 > 45 > 33 > 31 > 52 > 58) [27], ensuring one genotype per participant for risk estimation and preventing outcome misclassification due to multiple simultaneous detections.

### 2.4. Subgroup and Supportive Analyses

Age-specific positivity for DNA16/18 and mRNA16/18 was evaluated using predefined screening-relevant age bands (<25, 25–33, 34–69, and >69 years). Among DNA16/18-positive women, we further assessed mRNA16/18 as a triage marker for CIN2+. These analyses were motivated by prior Norwegian evidence showing that mRNA testing reduces false-positive findings relative to DNA testing and enhances risk discrimination in women with minor cytological abnormalities [14,15]. Therefore, the subgroup analyses aimed to contextualize mRNA performance within the genotypes prioritized in current triage guidelines and within age groups where overtreatment concerns are most relevant.

### 2.5. Referral Strategy Comparison (Simulation)

To estimate the potential impact of mRNA triage on downstream resource utilization, we compared two simplified referral strategies within this DNA-positive, low-grade cytology cohort:

(i) Refer all DNA-positive women to colposcopy, without additional stratification;
(ii) mRNA triage, referring only to mRNA-positive women and managing mRNA-negative women through routine surveillance with repeat HPV DNA testing at 12 months, with colposcopy referral reserved for women with persistent HPV positivity.

For each strategy, we calculated the referral rate and the number of colposcopies required per detected lesion (procedures per CIN2+ or CIN3+ among those referred). In addition, for the mRNA triage strategy, we quantified the number of CIN2+ and CIN3+ cases occurring in the mRNA-negative group, which would not be detected at baseline triage but would be expected to be identified at follow-up among women with persistent HPV positivity. These simulations were designed to reflect realistic clinical decision points and to quantify potential efficiency gains achievable with mRNA-guided triage while maintaining safety through surveillance of triage-negative women.

### 2.6. Data Sources and Quality Control

Data were extracted from laboratory information systems (HPV DNA, mRNA, cytology), colposcopy clinic databases, and pathology records. Cross-checks ensured internal consistency between test identifiers, collection dates, and outcome entries, and any discrepancies were resolved by chart review. Cytology terminology followed the 2014 Bethesda System, and data completeness was verified against the local registry before analysis [20].

### 2.7. Statistical Analysis

Test positivity and histological outcomes were summarized as counts and proportions. Diagnostic performance measures (sensitivity, specificity, PPV, NPV) were calculated using standard definitions with exact 95% confidence intervals. Relative risks (RRs) with 95% CIs were used to compare CIN2+ and CIN3+ risks between mRNA-positive and mRNA-negative women. Differences in proportions were assessed using χ^2^ tests or Fisher’s exact test when expected cell counts were <5.

Analyses were performed in IBM SPSS Statistics (version 29), with figures and referral simulations generated in R (version 4.3). Predefined contrasts included CIN2+ and CIN3+ risk by mRNA status, and, within DNA16/18-positive women, CIN2+ risk stratified by mRNA16/18 status.

For the simulated referral strategies, uncertainty in detection proportions was quantified using exact (Clopper–Pearson) 95% confidence intervals. These intervals were propagated to the metric “procedures per detected lesion” by mathematical inversion (1/p). No hypothesis testing was conducted for these scenarios, as the simulations represent deterministic outcomes based on the observed cohort.

### 2.8. Ethics

This study was approved by the Regional Committee for Medical and Health Research Ethics, North Norway, as a program evaluation project (REK Nord 203384). The study was conducted in accordance with institutional and national regulations and the Declaration of Helsinki. In line with Norwegian regulations, individual informed consent was not required for quality-assurance studies based on de-identified registry data.

## 3. Results

### 3.1. Study Population

During 2022–2024, 20,378 women underwent primary HPV DNA screening; 1243 (6.1%) were HPV DNA positive. Among HPV-positive women, 277 (22.3%) had low-grade cytology (ASC-US or LSIL) in reflex triage. Of these, 225 women had sufficient residual liquid-based cytology (LBC) material and a valid 7-type HPV E6/E7 mRNA result (PreTect HPV-Proofer 7) and were eligible for inclusion. The final analytic cohort comprised 175 women with complete follow-up and were included in the analysis (Figure 1).

All included cases were HPV DNA–positive on the cobas^®^ 4800 assay and were subsequently tested with the genotype-specific 7-type HPV E6/E7 mRNA assay. The analytic cohort comprised 115 women with ASC-US and 60 with LSIL cytology. The 7-type HPV mRNA assay was positive in 44.6% (78/175) of women—45.2% in the ASC-US group (52/115), and 43.3% in the LSIL group (26/60)—with no significant difference between cytology categories (χ^2^ = 0.06, *p* = 0.81). Overall, 55.4% (97/175) were mRNA negative. The overall prevalence of histologically confirmed CIN2+ and CIN3+ was 23.4% (41/175) and 9.7% (17/175), respectively. In the ASC-US subgroup, the prevalence of CIN2+ and CIN3+ was 22.6% (26/115) and 8.7% (10/115), respectively, whereas in the LSIL subgroup, the corresponding prevalences were 25.0% (15/60) and 11.7% (7/60) (Table 1).

### 3.2. Diagnostic Performance of mRNA Triage

When stratifying women by overall mRNA result—positive for any of the seven E6/E7 mRNA genotypes (HPV16, 18, 31, 33, 45, 52, or 58) versus mRNA-negative—the following diagnostic performance was observed:

- CIN2+: Among mRNA-positive women, 33.3% (26/78) had CIN2+, compared with 15.5% (15/97) among mRNA-negative women (relative risk [RR] = 2.16; 95% CI 1.23–3.78; χ^2^ = 7.70; *p* = 0.006). The corresponding test characteristics were: sensitivity 63.4%, specificity 61.2%, PPV 33.3%, and NPV 84.5% (Table 2A). Thus, 15 of 41 CIN2+ cases (36.6%) occurred in the mRNA-negative group and would not be detected at baseline triage, but would be expected to be identified at follow-up among women with persistent HPV positivity.
- CIN3+: For mRNA-positive women, the risk of CIN3+ was 14.1% (11/78) compared with 6.2% (6/97) among mRNA-negative women (RR = 2.27; 95% CI 0.88–5.87; χ^2^ = 3.07; *p* = 0.08). Sensitivity, specificity, PPV, and NPV were 64.7%, 57.6%, 14.1%, and 93.8%, respectively (Table 2B). Accordingly, 6 of 17 CIN3+ cases (35.3%) occurred in the mRNA-negative group and would not be detected at baseline triage, but would be expected to be identified at follow-up among women with persistent HPV positivity.

### 3.3. Genotype-Specific Risk Stratification

Among mRNA-positive women, genotype-specific risks for CIN2+ showed a clear stepwise gradient. The positive predictive value (PPV) was highest for HPV33, HPV18, HPV16, and HPV31, and lower for HPV45, HPV58, and HPV52. mRNA-negative women had a low CIN2+ risk (15.5%).

For CIN3+, HPV33 demonstrated the highest PPV (50%), followed by intermediate values for HPV16, HPV18, and HPV31, while HPV45, HPV52, and HPV58 were rarely or not associated with CIN3+. Six CIN3+ cases were observed among mRNA-negative women (6.2%). Although denominators for HPV33 and HPV18 were small (*n* = 6 each), the consistent pattern of risk (33 > 18 > 16 > 31 ≫ 45/58/52) underscores the discriminatory value of genotype-specific mRNA triage (Table 3).

### 3.4. Co-Infections Detected by the 7-Type HPV mRNA Assay

Among mRNA-positive women (*n* = 78), co-infections (≥2 genotypes) were detected in 11.5% (9/78), corresponding to 5.1% of the total cohort (9/175). Most co-infection patterns were rare and occurred as single observations, with HPV16 + HPV58 being the only combination observed more than once (2/78; 2.6%). The full distribution of co-infection patterns is presented in Table 4. Because the numbers within specific co-infection combinations were very small, separate statistical analyses of individual co-infection patterns were not performed.

### 3.5. Age-Specific Comparison of HPV16/18 DNA and mRNA Detection, and Triage Within HPV16/18 DNA-Positive Women

Across all age groups, detection of transcriptionally active HPV16/18 infections by mRNA testing was approximately 30–36% lower than detection of viral presence by DNA testing (overall 16.0% [28/175] vs. 24.0% [42/175]), indicating that a substantial proportion of HPV16/18 DNA-positive cases lacked detectable E6/E7 mRNA expression (χ^2^ = 3.95, *p* = 0.047). By age group, mRNA versus DNA positivity rates were 14.7% vs. 20.6% among women aged 25–33 years, 18.6% vs. 28.9% in those aged 34–69 years, and 0% vs. 0% in the <25 and >69 age groups (Table 5).

**Figure 2:**
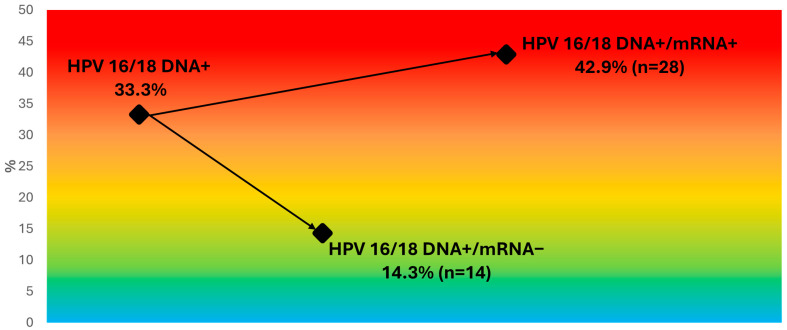
Risk stratification among HPV16/18 DNA–positive women. The figure illustrates how E6/E7 mRNA testing (PreTect HPV-Proofer 7) separates HPV16/18 DNA–positive women with low-grade cytology (ASC-US/LSIL) into high- and low-risk subgroups for CIN2+. Diamonds indicate observed CIN2+ proportions, and arrows depict the divergence from the overall DNA-based risk toward the mRNA-defined strata. The background gradient represents increasing CIN2+ risk from cool (low) to warm (high) colors. This visualization highlights the added discriminatory power of mRNA16/18 triage within the HPV16/18 DNA-positive group.

As shown in Table 4, mRNA positivity for HPV16/18 was consistently lower than DNA positivity across age groups. Among women who were HPV16/18 DNA-positive (*n* = 42), the mRNA assay further stratified risk: 42.9% (12/28) of mRNA16/18-positive women had CIN2+, compared with 14.3% (2/14) of those who were mRNA16/18-negative (relative risk ≈ 3.0; 95% CI 0.79–11.3; Fisher’s exact *p* = 0.10) (Table 6, Figure 2).

### 3.6. Simulated Referral Strategies and Procedural Yield

When comparing immediate referral of all HPV DNA–positive ASC-US/LSIL cases with an mRNA-triage strategy (referring mRNA-positive and monitoring mRNA-negative women), the overall referral rate would decline from 100% to 44.6% (95% CI: 38.0–52.0). For CIN2+, the number of colposcopies required per detected lesion would decrease from 4.27 (95% CI: 3.29–5.78) to 3.00 (95% CI: 2.23–4.29). For CIN3+, the corresponding values would decrease from 10.3 (95% CI: 6.58–17.2) to 7.1 (95% CI: 4.18–13.7) (Table 7).

Compared with referring all HPV DNA–positive ASC-US/LSIL cases directly to colposcopy, the mRNA-triage approach reduced overall referrals by 55% and improved referral efficiency, lowering the number of colposcopies required per detected CIN2+ and CIN3+ lesion by 30% and 31%, respectively.

### 3.7. Summary of Principal Findings

In HPV DNA–positive women with ASC-US/LSIL cytology, genotype-specific E6/E7 mRNA triage demonstrated:

(i) approximately 2-fold higher risk separation between test-positive and test-negative women for CIN2+ and CIN3+,
(ii) the highest genotype-specific risks for HPV33, HPV18, HPV16, and HPV31, and
(iii) a substantial reduction in referral rates and procedures per detected CIN2+ or CIN3+ lesion compared with referring all DNA-positive low-grade cases (Table 1, Table 2, Table 3, Table 4, Table 5, Table 6 and Table 7).

## 4. Discussion

### 4.1. Principal Findings

In this real-world cohort of HPV DNA–positive women with ASC-US/LSIL cytology (N = 175), genotype-specific E6/E7 mRNA triage using the 7-type PreTect HPV-Proofer 7 assay improved clinical discrimination between transient and clinically significant infections. The assay yielded an overall mRNA positivity rate of 44.6%, similar across cytology categories. Compared with HPV DNA results alone, mRNA triage increased the separation in CIN2+/CIN3+ risk between test-positive and test-negative women, identifying HPV33, HPV18, HPV16, and HPV31 as the genotypes most strongly associated with high-grade lesions. Importantly, mRNA-negative women—constituting 55.4% of the cohort—formed a sizeable low-risk group suitable for surveillance rather than immediate referral.

In the referral simulation, an mRNA-guided strategy would reduce baseline referrals by approximately half and decrease the number of colposcopies per detected CIN2+/CIN3+ by roughly one-third compared with referring all DNA-positive low-grade cases. This gain in efficiency should be interpreted in light of the fact that 15 CIN2+ and 6 CIN3+ cases occurred among mRNA-negative women and would not be detected at baseline triage, but would be expected to be identified at 12-month follow-up among women with persistent HPV positivity.

### 4.2. Context with Prior Evidence

The present findings align with and extend a substantial body of evidence demonstrating that HPV E6/E7 mRNA testing provides higher specificity and greater clinical efficiency than cytology or HPV DNA testing for the triage of women with minor cytological abnormalities.

Earlier Norwegian and Nordic studies using the 5-type PreTect HPV-Proofer assay have shown that, in women with ASC-US/LSIL cytology, mRNA-based triage yields lower test positivity and fewer referrals than DNA-based strategies, while maintaining comparable detection of high-grade disease. This supports the higher specificity of E6/E7 mRNA testing in cytology-based screening settings and provides an evidence base for extending mRNA triage to HPV DNA–positive cohorts, as evaluated here with a 7-type assay [14,15,28].

Population-based analyses further reinforce these outcomes. In Norwegian registry data among women aged 25–33 years with ASC-US/LSIL, an mRNA-based triage pathway demonstrated markedly lower test positivity and substantially reduced downstream health-care utilization compared with DNA-based triage, while maintaining equivalent long-term protection against cervical cancer over six years of follow-up [25]. These programmatic advantages are particularly relevant in the era of primary HPV DNA screening, where a large proportion of women are DNA-positive and require secondary triage. Within this group, DNA testing offers no additional risk stratification, and cytology alone remains imperfect. In this context, E6/E7 mRNA testing provides a biologically grounded and operationally efficient triage layer by identifying transcriptionally active infections most likely to reflect clinically significant disease.

These observations have been replicated outside the Nordic region. Studies from Italy, Greece, and North Macedonia further validated PreTect HPV-Proofer 5 as a reliable molecular biomarker for risk stratification among HPV DNA–positive women [29,30,31,32]. Several of these investigations also demonstrated the utility of E6/E7 mRNA testing in post-treatment follow-up, where persistent mRNA positivity predicted residual or recurrent CIN2/3+, while mRNA negativity was strongly associated with lesion regression or successful treatment outcomes [33,34]. Notably, Bruno et al. showed that mRNA-negative status predicted spontaneous regression of CIN2 lesions in high-risk HPV–positive women, particularly among younger women with high HPV prevalence, underscoring the prognostic value of mRNA as a marker of lesion dynamics and viral clearance [33].

Additional evidence from Southern Europe further supports the specificity advantage of HPV E6/E7 mRNA–based triage in women with low-grade cytological abnormalities. In a large Italian cohort of women aged 21–24 years with ASC-US or LSIL, Frega et al. directly compared high-risk HPV DNA testing, p16/Ki-67 dual staining (CINtec^®^ PLUS), and HPV E6/E7 mRNA testing in relation to histologically confirmed CIN2–3 [35]. While HPV DNA testing demonstrated very high sensitivity for CIN2+—approaching 100% in ASC-US and remaining high in LSIL (~93%)—its specificity was extremely low (17–23%), reflecting the high prevalence of transient infections in this age group. In contrast, HPV E6/E7 mRNA testing achieved substantially higher specificity (≈81–88%) while maintaining sensitivity for CIN2 and CIN3, outperforming HPV DNA testing and demonstrating equal or superior specificity compared with p16/Ki-67 dual staining. This advantage was most pronounced in LSIL, where mRNA specificity for CIN2 and CIN3 (~88% and ~81%, respectively) markedly exceeded that of p16/Ki-67 (~62% and ~49%). Importantly, the authors concluded that mRNA-based triage could safely replace immediate colposcopy with surveillance in mRNA-negative women, thereby reducing overtreatment in young women with a high likelihood of spontaneous regression.

More recent population-based research employing the 7-type PreTect HPV-Proofer 7 assay in the context of HPV DNA primary screening further confirmed these advantages. In a head-to-head comparison, mRNA triage demonstrated higher specificity and positive predictive value than liquid-based cytology while maintaining similar CIN2+ sensitivity, resulting in fewer colposcopies per detected high-grade lesion [16].

Beyond Europe, Proofer 7 has shown consistent performance in global settings. In South Africa, where HPV prevalence and HIV co-infection are high, genotype-specific mRNA testing effectively discriminated clinically meaningful infections in a high-risk population [36]. Likewise, in Mexico, evaluation of Proofer 7 in a referral population identified through ASC-US+ cytology confirmed its ability to reduce unnecessary colposcopy referrals while preserving high-grade disease detection [37].

Collectively, evidence across clinical cohorts, population registries, international studies, and post-treatment follow-up demonstrates that genotype-specific E6/E7 mRNA detection reliably identifies transcriptionally active, clinically significant HPV infections across diverse screening algorithms, epidemiological settings, and risk profiles. The cumulative data support mRNA triage as a biologically grounded and operationally efficient complement to extended DNA genotyping in contemporary cervical cancer prevention programs.

### 4.3. Why Genotype-Specific mRNA Matters in a DNA-Positive, Low-Grade Group

By design, HPV DNA testing provides no further discrimination once all women in the analytic set are DNA-positive; subsequent risk differentiation must therefore rely on viral genotype and on the biological activity of the infection. Extended DNA genotyping, as now recommended in the 2025 Norwegian guidelines, recognizes this heterogeneity by differentiating risk according to genotype, particularly for HPV16, HPV18, HPV31, HPV33, HPV45, HPV52, and HPV58 [10]. These seven genotypes are precisely those included in the PreTect HPV-Proofer 7 assay, reflecting their established contribution to >90% of cervical cancers worldwide.

However, even within these high-risk genotypes, DNA positivity alone does not distinguish between transient infection and biologically active lesions undergoing oncogenic transformation. The present study demonstrates that genotype-specific mRNA detection adds clinically meaningful stratification beyond what DNA genotyping alone provides. Among DNA16/18-positive women, mRNA testing further separated high- and low-risk subgroups, tripling the observed CIN2+ risk between mRNA-positive and mRNA-negative women. More broadly across all types, we observed a clear stepwise gradient in genotype-specific PPVs, with the highest risks for HPV33, HPV18, HPV16, and HPV31—mirroring larger population-based findings where mRNA PPVs exceeded DNA-based risk estimates for the same genotypes [16].

This reinforces the principle that genotype determines the potential for disease, but mRNA expression identifies biologically active disease. In Sørbye et al., genotype-specific mRNA triage outperformed both cytology and extended DNA genotyping in risk discrimination and referral efficiency, reducing unnecessary colposcopies while maintaining CIN2+/CIN3+ detection [16]. The present findings corroborate that mRNA triage remains valuable even within a modern DNA genotyping framework and provides an additional, biologically grounded filter to avoid over-referral of women whose infections are not transcriptionally active.

Together, these results support the complementary roles of extended DNA genotyping and mRNA triage: DNA identifies who is infected and with which genotype, while mRNA determines which infections are clinically relevant. In a DNA-positive, low-grade cytology population, this layered approach substantially refines risk stratification and improves the efficiency of colposcopy use.

### 4.4. Refining HPV16/18 Risk: DNA–mRNA Differentiation and Implications for Targeted Triage

Across the screening age range, mRNA16/18 positivity was consistently 30–36% lower than DNA16/18 positivity (overall 16.0% vs. 24.0%; *p* = 0.03), demonstrating that a substantial proportion of HPV16/18 DNA-positive infections lacked detectable E6/E7 transcriptional activity. This underscores the distinction between viral presence and biologically active infection, even for the genotypes with the highest oncogenic potential.

Within the subgroup of HPV16/18 DNA-positive women (*n* = 42), mRNA16/18 further enriched CIN2+ risk: 42.9% among mRNA-positive women versus 14.3% among mRNA-negative women (RR ≈ 3.0; 95% CI 0.80–11.1; *p* = 0.09). Despite the modest sample size, this gradient highlights the ability of mRNA testing to discriminate clinically meaningful infections from transient viral presence, supporting its use as a refinement step following partial DNA genotyping.

This is particularly relevant for younger women (<35 years), who exhibit the highest HPV DNA prevalence and elevated CIN2+ detection, yet also experience high rates of spontaneous CIN2 regression [38]. While overtreatment in this age group carries significant reproductive risks, the balance is complicated by the fact that HPV16, HPV18, and HPV45 account for most cervical cancers diagnosed in young women and are associated with more rapid progression compared with other oncogenic genotypes [18,19]. Genotype-specific mRNA detection, therefore, offers dual value: (i) identifying transcriptionally active infections among these high-risk genotypes, and (ii) providing reassurance for conservative management when mRNA is negative, particularly for younger women and in CIN2 cases where surveillance is considered.

These observations are consistent with population-level and young-women cohort data showing that mRNA triage maintains sensitivity while substantially improving specificity over DNA- and cytology-based approaches [16,37]. Together, they support a combined strategy in which DNA genotyping identifies women infected with the fastest-progressing, highest-risk genotypes, while mRNA testing clarifies which of these infections are transcriptionally active and clinically actionable.

### 4.5. Clinical and Programmatic Implications

For clinical services triaging HPV DNA–positive women with ASC-US/LSIL cytology, incorporating genotype-specific E6/E7 mRNA testing offers several programmatically meaningful advantages:

1. Substantial reduction in referrals: mRNA-based triage would reduce colposcopy referrals by ~55%, easing pressure on overstretched colposcopy services.
2. Improved diagnostic efficiency: Fewer procedures are required per detected CIN2+/CIN3+ lesion, reflecting a more efficient allocation of diagnostic resources.
3. Targeted evaluation of women at highest immediate risk: mRNA positivity identifies infections with transcriptionally active oncogene expression, allowing diagnostic work-ups to focus on women most likely to harbor CIN2+ or rapidly progressing lesions.
4. Safe surveillance of low-risk individuals: mRNA-negative women—over half of the cohort—formed a low-risk group with a low CIN2+/CIN3+ yield, supporting surveillance rather than immediate referral. This is particularly important for younger women (<35), in whom CIN2 frequently regresses and overtreatment poses reproductive risks.

These clinical gains parallel those observed when mRNA triage replaces cytology in broader HPV DNA–positive populations [16], reaffirming mRNA’s role as a more specific, objective, and biologically grounded triage modality. Importantly, the benefits persist even within the context of extended DNA genotyping, where mRNA adds discriminatory power by distinguishing transcriptionally active infections from transient viral presence. As many screening programs transition toward genotype-based management, mRNA triage represents a complementary refinement step that can help balance cancer prevention with the imperative to minimize unnecessary interventions.

### 4.6. Strengths and Limitations

This study has several strengths. Its real-world design reflects routine clinical practice in an organized screening program, enhancing applicability to contemporary screening pathways. The analytic cohort was restricted to women with sufficient follow-up to classify outcomes, and linkage to cytology, HPV testing, and histology enabled comprehensive outcome ascertainment among included participants. The use of genotype-specific E6/E7 mRNA testing enabled detailed, type-resolved risk estimates in a setting where only partial HPV16/18 versus “other” DNA genotyping was available during the study period. These mRNA-based risk profiles are directly relevant to current triage frameworks that now incorporate extended DNA genotyping, as they illustrate how transcriptional activity varies across the same oncogenic genotypes that are prioritized in modern algorithms. The integration of matched DNA and mRNA results within a clearly defined ASC-US/LSIL population therefore, provides a robust assessment of how mRNA triage performs in the exact clinical context for which it is intended—refining risk among HPV DNA–positive women and improving discrimination beyond DNA signals alone.

Several limitations warrant consideration. The study was conducted at a single center, which may limit representativeness, and the modest sample size—particularly within specific genotype strata such as HPV33 and HPV18 (*n* = 6 each)—results in wide confidence intervals around type-specific predictive values that should be interpreted with caution. In addition, when more than one E6/E7 mRNA genotype was detected, we assigned a single index genotype using a predefined hierarchy based on established oncogenic potential; this approach may oversimplify co-infections, could influence type-specific risk estimates, and does not allow assessment of potential synergistic effects between genotypes. The complete distribution of co-infections is provided in a dedicated table, but counts within individual co-infection patterns were small, precluding meaningful risk analyses by specific combinations. Follow-up time (maximum about 3.5 years) was shorter than in some larger Norwegian and international cohorts with 5–10 years of follow-up, and our data, therefore, cannot fully address very long-term safety after a negative mRNA triage result.

Histological verification was determined by routine clinical management based on HPV DNA and cytology rather than by mRNA results. Consequently, not all women underwent biopsy: some were managed with surveillance, and some may have had colposcopy without biopsy if no lesion was visible. Women without histology were retained in the analytic cohort only if they had a documented HPV-negative follow-up test within the observation period; women lacking both histology and HPV-negative follow-up were excluded. This approach may introduce verification bias and some degree of outcome misclassification, which could affect estimates of genotype-specific risks and test performance. Individual-level covariates such as HPV vaccination status, smoking, and reproductive history were not available, precluding adjustment for potential confounders and limiting generalizability, particularly to fully vaccinated cohorts. Finally, the mRNA assay targets seven genotypes and therefore does not capture all oncogenic types included in the primary HPV DNA screening test; CIN2+ caused by non-targeted types will, by design, be mRNA negative, and our findings cannot be directly extrapolated to triage strategies that rely solely on broader DNA-based genotyping that operates under different screening intervals, cytology infrastructure, or adherence to recommended follow-up.

### 4.7. Future Directions

Further research should build on these findings in several ways. First, larger multi-center studies are needed to refine genotype-specific risk estimates and narrow confidence intervals, particularly for less prevalent types such as HPV33 and HPV18. Such pooled analyses would strengthen the evidence base for incorporating genotype-resolved mRNA markers into national triage algorithms.

Second, the development of integrated risk-based models that combine mRNA genotype results with age, prior screening history, vaccination status, and cytological or colposcopic findings could enable more individualized management strategies. These models may be especially valuable for younger women, in whom distinguishing transient CIN2 from biologically active, progressive lesions is critical to avoid overtreatment.

Third, evaluation of mRNA-based triage in self-sampling pathways represents an important opportunity for programmatic innovation. Because mRNA assays are objective, molecular, and compatible with automated laboratory workflows, they may enhance the scalability, reproducibility, and accessibility of population screening—particularly in underscreened groups or settings with limited cytology infrastructure.

Together, these directions can help integrate genotype-specific mRNA testing more fully into modern HPV screening frameworks, supporting both precision triage and more efficient use of healthcare resources.

## 5. Conclusions

In HPV DNA-positive women with ASC-US/LSIL cytology, genotype-specific HPV E6/E7 mRNA triage provides a level of specificity and biological risk discrimination that neither cytology nor DNA testing alone can achieve. By identifying transcriptionally active infections, the assay substantially improved risk separation for CIN2+ and CIN3+, clearly distinguishing high-risk genotypes—most notably HPV33, HPV18, HPV16, and HPV31—from transient or clinically insignificant infections. More than half of the cohort was mRNA-negative, forming a low-risk group appropriate for surveillance rather than immediate referral.

A genotype-specific, mRNA-guided referral strategy would reduce colposcopy referrals by approximately 55% and decrease the number of procedures required per detected high-grade lesion by one-third, representing a major gain in diagnostic efficiency. These benefits are especially relevant in younger women, in whom CIN2 is common but frequently regresses, and in whom avoiding unnecessary treatment is a clinical priority.

Importantly, the added value of mRNA triage persists even within modern extended DNA genotyping frameworks, where mRNA provides the crucial biological distinction between viral presence and active oncogene expression. Together, these findings support the integration of genotype-aware mRNA triage into contemporary cervical screening programs to optimize the balance between early detection, avoidance of overtreatment, and efficient use of colposcopy resources.

## Figures and Tables

**Table 1:** Cohort characteristics, cytology distribution, and mRNA test positivity (N = 175).

Characteristic	*n*/N (%)
Cytology	
ASC-US	115/175 (65.7)
LSIL	60/175 (34.3)
Histology outcome	
CIN2+ prevalence (overall)	41/175 (23.4)
ASC-US subgroup	26/115 (22.6)
LSIL subgroup	15/60 (25.0)
CIN3+ prevalence (overall)	17/175 (9.7)
ASC-US subgroup	10/115 (8.7)
LSIL subgroup	7/60 (11.7)
mRNA (7-type) positive	78/175 (44.6)
ASC-US subgroup	52/115 (45.2)
LSIL subgroup	26/60 (43.3)

Abbreviations: ASC-US, atypical squamous cells of undetermined significance; LSIL, low-grade squamous intraepithelial lesion; CIN, cervical intraepithelial neoplasia.

**Table 2:** (**A**) Diagnostic performance of 7-type HPV mRNA triage for detection of CIN2+ among HPV DNA–positive women with ASC-US/LSIL (N = 175). (**B**) Diagnostic performance of 7-type HPV mRNA triage for detection of CIN3+ among HPV DNA–positive women with ASC-US/LSIL (N = 175).

(**A**)				
**mRNA Result**	**CIN2+ (*n*)**	**≤CIN1 (*n*)**	**Total (*n*)**	**Predictive Value**
Positive	26	52	78	PPV 33.3%
Negative	15	82	97	NPV 84.5%
Total	41	134	175	-
(**B**)				
**mRNA Result**	**CIN3+ (*n*)**	**≤CIN2 (*n*)**	**Total (*n*)**	**Predictive Value**
Positive	11	67	78	PPV 14.1%
Negative	6	91	97	NPV 93.8%
Total	17	160	175	-

(**A**) Performance metrics: Sensitivity 63.4%, Specificity 61.2%. Risk separation: CIN2+ risk 33.3% (mRNA+) vs. 15.5% (mRNA−). Note: ≤CIN1 includes normal histology (NILM) and CIN1 findings. CIN2+ includes CIN2, CIN3, adenocarcinoma in situ, and invasive carcinoma. (**B**) Performance metrics: Sensitivity 64.7%, Specificity 57.6%. Risk separation: CIN3+ risk 14.1% (mRNA+) vs. 6.2% (mRNA-), RR 2.27. Note: ≤CIN2 includes normal histology (NILM), CIN1, and CIN2 findings. CIN3+ includes CIN3, adenocarcinoma in situ, and invasive carcinoma.

**Table 3:** Genotype-specific positive predictive values (PPVs) for CIN2+ and CIN3+ with 7-type HPV mRNA triage.

mRNA Genotype	N (Total Positive)	CIN2+ (*n*, PPV%)	CIN3+ (*n*, PPV%)
16	22	9 (40.9%)	4 (18.2%)
18	6	3 (50.0%)	1 (16.7%)
31	20	7 (35.0%)	2 (10.0%)
33	6	4 (66.7%)	3 (50.0%)
45	5	1 (20.0%)	1 (20.0%)
52	11	1 (9.1%)	0 (0.0%)
58	8	1 (12.5%)	0 (0.0%)
mRNA-negative	97	15 (15.5%)	6 (6.2%)

Note: Small denominators for HPV33 and HPV18 (*n* = 6 each) imply wide confidence intervals, but the observed gradient in risk (33 > 18 > 16 > 31 ≫ 45/58/52) is consistent.

**Table 4:** Distribution of co-infections detected by the 7-type HPV E6/E7 mRNA assay among mRNA-positive women (*n* = 78).

mRNA Genotypes Detected (Co-Infection Pattern)	N (%)
HPV16 + HPV58	2/78 (2.6)
HPV33 + HPV52	1/78 (1.3)
HPV16 + HPV52	1/78 (1.3)
HPV31 + HPV33	1/78 (1.3)
HPV18 + HPV31	1/78 (1.3)
HPV18 + HPV52	1/78 (1.3)
HPV18 + HPV45 + HPV58	1/78 (1.3)
HPV16 + HPV18 + HPV52 + HPV58	1/78 (1.3)

Note: Co-infections (≥2 genotypes) were detected in 9/78 (11.5%) of mRNA-positive women (5.1% of the full cohort, N = 175). Counts within individual co-infection patterns were small.

**Table 5:** Age-specific HPV16/18 positivity by DNA and mRNA testing (N = 175).

Age Group (Years)	HPV16/18 DNA *n* pos/Total (% Positive)	HPV16/18 mRNA *n* pos/Total (% Positive)	Relative Reduction in Positivity (mRNA vs. DNA)
<25	0/9 (0.0%)	0/9 (0.0%)	—
25–33	14/68 (20.6%)	10/68 (14.7%)	−28.6%
34–69	28/97 (28.9%)	18/97 (18.6%)	−35.7%
>69	0/1 (0.0%)	0/1 (0.0%)	—
Total	42/175 (24.0%)	28/175 (16.0%)	−33.3%

**Table 6:** Performance of mRNA16/18 triage for CIN2+ among HPV16/18 DNA–positive women (*n* = 42).

mRNA16/18 Result	CIN2+ (*n*)	≤CIN1 (*n*)	Total (*n*)	Predictive Value
Positive	12	16	28	PPV 42.9%
Negative	2	12	14	NPV 85.7%
Total	14	28	42	-

Performance metrics: Sensitivity 85.7%, Specificity 42.9%, Relative Risk (CIN2+ mRNA+ vs. mRNA−) ≈ 3.0 (95% CI 0.79–11.3; *p* = 0.10, Fisher’s exact test). Referral efficiency: Referring all HPV16/18 DNA-positive women required 3.00 colposcopies per CIN2+ detected, whereas using mRNA16/18 triage reduced this to 2.33 colposcopies per CIN2+, representing a 22% improvement in efficiency.

**Table 7:** Comparison of simulated referral strategies among HPV DNA–positive women with ASC-US/LSIL cytology (N = 175).

Referral Strategy	Referrals (*n*, %)	CIN2+ Detected	Colposcopies per CIN2+	CIN3+ Detected	Colposcopies per CIN3+
Refer all HPV DNA-positive with ASC-US/LSIL to colposcopy	175 (100%)	41	4.27	17	10.3
mRNA triage (refer only to mRNA-positive among HPV DNA-positive with ASC-US/LSIL)	78 (44.6%)	26	3.00	11	7.1

## Data Availability

The data presented in this study are available on reasonable request from the corresponding author. The data are not publicly available due to privacy and ethical restrictions in accordance with Norwegian data protection regulations.

## Abbreviations

The following abbreviations are used in this manuscript:

ASC-US	Atypical squamous cells of undetermined significance
CI	Confidence interval
CIN	Cervical intraepithelial neoplasia
CIN2+	Cervical intraepithelial neoplasia grade 2 or worse
CIN3+	Cervical intraepithelial neoplasia grade 3 or worse
DNA	Deoxyribonucleic acid
HPV	Human papillomavirus
HSIL	High-grade squamous intraepithelial lesion
LBC	Liquid-based cytology
LSIL	Low-grade squamous intraepithelial lesion
mRNA	Messenger ribonucleic acid
NPV	Negative predictive value
PPV	Positive predictive value
REK	Regional Committee for Medical and Health Research Ethics (Norway)
RR	Relative risk
